# Thyroid: From Genes to the Disease

**DOI:** 10.2174/138920211798120817

**Published:** 2011-12

**Authors:** Katja Zaletel

This issue of Current genomics is devoted to a broad spectrum of thyroid disorders which occur in the population with a high frequency and represent an important clinical problem to thyroid specialists all over the world. Understanding the disease pathophysiology represents an important tool for clinicians confronting various diagnostic and therapeutic challenges in managing patients. In the last decade we have been facing a tremendous advance in our knowledge and numerous evidence of a susceptibility to various thyroid disorders. All this is helping us to understand the mechanisms, offering promises for prevention and even having an impact on the way we treat patients. It is now clear that a certain genotype in combination with a proper environmental trigger is crucial for the disease development, but several steps from this point to the clinical disease remain to be elucidated. This special focus issue summarizes current knowledge of thyroid genetics, emphasizing the importance of genetic contribution to a distinctive clinical outcome in thyroid autoimmune disease and in thyroid neoplasm. 

In thyroid autoimmune disease, the evidence on genetic susceptibility obtained by epidemiological, family and twin studies has been confirmed using the whole genome and candidate gene approaches. Those studies helped to uncover major candidate genes, some of them being thyroid specific, while others are being involved in the immune regulation. Brand and Gough comprehensively reviewed immunogenetic mechanisms of thyroid autoimmunity and genotyping technologies, illustrating both historical and recent aspects as well as future challenges. In spite of shared genetic predisposal factors, different immunogenetic pathways lead to specific subtypes of thyroid autoimmunity, such as Graves’ disease, Graves’ ophthalmopathy and Hashimoto’s thyroiditis. Detailed up-date on the current knowledge of genetic markers associated with Graves’ disease, presented by Ploski and colleagues, also explains a functional significance of observed associations and genotype-phenotype correlations. Immunogenetics of Graves’ ophthalmopathy, being clinically detected in up to 50% of patients with Graves’ disease, is reviewed by Khalilzadeh and colleagues, emphasizing both genetic background and putative events in immune activation in the orbit. In contrast to Graves’ disease, where humoral immune response predominates, cell-mediated immune response is characteristic for Hashimoto’s thyroiditis. Zaletel and Gaberšček provided an extensive overview of the possible triggers as well as putative mechanisms leading to the clinical disease. 

When discussing thyroid tumors, thyroid nodule is a frequent finding in adults, especially in those undergoing thyroid ultrasound examinations. Taking into account a high prevalence and most frequently a benign nature, diagnosing of thyroid carcinoma seems like looking for a needle in a haystack. Although fine needle aspiration biopsy is a convenient and the best non-surgical diagnostic tool for distinguishing between benign and malignant thyroid lesion, its accuracy varies with the histological subtype of thyroid nodule and in a high percentage of specimen indeterminate cytology is reported. The article of Cerutti is a detailed review of all different approaches used in recent years to overcome a problem of differentiation of benign from malignant thyroid tumors, emphasizing molecular markers that may improve diagnostic accuracy and help to avoid unnecessary treatment of benign tumors. Detection of specific genetic alterations in thyroid cancer seems to be useful not only for the diagnosis, but also for the prognosis of the disease. While the author Gomez Saez described different genetic abnormalities focusing on differentiated histological subtypes of thyroid carcinoma, Soares and co-workers reviewed the most relevant genetic alterations in aggressive poorly differentiated and undifferentiated thyroid carcinomas. The current knowledge and approaches in medullary thyroid carcinoma are summarized by Taccaliti and colleagues, focusing on the central role of *RET* proto-oncogene in management of patients and their families. In the recent years, the rapidly growing knowledge of the molecular pathways in thyroid cancer enabled the development of new therapeutic agents. Antonelli and co-workers contributed a timely review of this relevant topic, providing both an overview on molecular pathways being explored as therapeutic targets and detailed information on new targeted therapies for thyroid carcinoma. 

Hopefully, the selected topics of this thematic issue will offer critically summarized and timely information from the field of thyroid pathology relevant to physicians, clinical researchers and basic scientists. I would especially like to thank the reviewers for their valuable time, comments, and suggestions that contributed to an even better quality of the reviews. Finally, let me thank Editor-in-Chief, Dr. Christian Neri, and his co-workers for the trust and support during the process of preparing this special focus issue.

